# “Not everything that can be counted counts” in ethanol toxicological results: an antemortem and postmortem technical interpretation focusing on driving under the influence

**DOI:** 10.1093/fsr/owae023

**Published:** 2024-04-02

**Authors:** Ricardo Jorge Dinis-Oliveira

**Affiliations:** Associate Laboratory i4HB - Institute for Health and Bioeconomy, University Institute of Health Sciences - CESPU, Gandra, Portugal; UCIBIO - Research Unit on Applied Molecular Biosciences, Translational Toxicology Research Laboratory, University Institute of Health Sciences 1H-TOXRUN, IUCS-CESPU, Gandra, Portugal; Department of Public Health and Forensic Sciences and Medical Education, Faculty of Medicine, University of Porto, Porto, Portugal; FOREN – Forensic Science Experts, Lisbon, Portugal

**Keywords:** ethanol, alcohol, postmortem diffusion, endogenous, putrefaction, blood, urine, vitreous humour, interpretation, biomarkers

## Abstract

Ethanol blood analysis is the most common request in forensic toxicology, and some studies point to positive results in approximately one-third of all unnatural deaths. However, distinguishing sober deaths from drunk deaths is not as simple as it may seem. This technical, clinical, and forensic interpretation is proposed to interpret the ethanol toxicological results, discussing several artefacts and pitfalls that must be considered, namely focusing on driving under the influence. This work is presented with a practical and objective approach, aiming to alleviate the complexities associated with clinical, physiological, pathophysiological, and toxicological aspects to enhance comprehension, practicality, and applicability of its content, especially to courts. Particularly the physical integrity of the body, the postmortem interval, putrefactive signs, anatomic place of blood collection, alternative samples such as vitreous humour and urine, the possibility of postmortem redistribution, the inclusion of preservatives in containers, and optimal temperature conditions of shipment are among some of the aspects to pay attention. Although several biomarkers related to postmortem microbial ethanol production have been proposed, their translation into forensic routine is slow to be implemented due to the uncertainties of their application and analytical difficulties. Specifically, in the interpretation of ethanol toxicological results, “not everything that can be counted counts and not everything that counts can be counted” (attributed to Albert Einstein).

## Introduction

According to the World Health Organization and other studies, driving under the influence of psychoactive substances is a significant public health problem worldwide [1, 2]. Ethanol is among the most frequently occurring toxicological substances in antemortem and postmortem toxicological cases related to road driving accidents. The legal framework for driving under the influence of psychoactive substances varies globally [3]. Still, three approaches are commonly employed: (i) the zero-level tolerance laws, which deem road driving illegal with any detectable amount of substances in the body; (ii) the laws favouring the state of influence (or homologous designations) which consider road driving illegal when the capabilities to do so are impaired by the action of psychoactive substances, and (iii) the laws setting minimum cut-off values for concentrations, making road driving illegal when specific blood levels of psychoactive substances are exceeded. For ethanol, in Portugal, the third model is applied [3], but the interpretation of toxicological results is challenging specially postmortem, due to several artefacts. Indeed, ethanol concentration may represent intake prior to death, postmortem formation and/or redistribution, or a combination of the two possibilities. Therefore, complementary analyses, such as concentration ratios among different samples collected from the same victim; analyses of other putrefactive alcohols in blood, such as 1-propanol and 1-butanol; and analyses of ethanol metabolites of nonoxidative pathways, such as ethylglucuronide (EtG), ethylsulfate (EtS), and phosphatidylethanol (PEth); and the urinary metabolites of serotonin 5-hydroxytryptophol/5-hydroxyindoleacetic acid (5-HTOL/5-HIAA), which are present at comparatively higher concentrations in central blood than in peripheral blood due to diffusion or formation, increase the accuracy of interpretation and support the hypothesis that the ethanol detected concentration is partly or entirely caused by postmortem formation. This pragmatic review is designed to interpret the influence that ethanol may have on driving and aims to discuss several aspects that must be considered in the interpretation of toxicological results in either living individuals or cadavers. This reflection is particularly critical since recent data may bring into question much of what has been performed. Indeed, in 24% of ethanol-positive autopsies, some postmortem formation occurred, and postmortem ethanol was also present in the urine and vitreous humour more frequently than expected, especially at lower ethanol levels [4].

## Methods

A thorough exploration was undertaken across various databases, encompassing PubMed (US National Library of Medicine), Web of Science, Embase, SCOPUS, and Google Scholar, to attain comprehensive cross-disciplinary coverage. No date limit was applied. This technical analysis was supplemented by referencing Portuguese legislation pertaining to this matter, including (i) Decree-Law No. 44/2005, dated 23 February—which regulates the Portuguese Road Code; (ii) Law No. 72/2013, dated 3 September—amends the Portuguese Road Code; (iii) Law No. 18/2007, dated 17 May—approves the regulation of driving under the influence of alcohol and other psychotropic substances; (iv) Ordinance No. 902-A/2007, dated 13 August—approved the fees applicable when driving under the influence of alcohol and other psychotropic substances; and (v) Ordinance No. 902-B/2007, dated 13 August—approves the materials for determining alcohol presence in expired air and for collecting and transporting biological samples to quantify blood alcohol concentration (BAC) or detect psychotropic substances in the blood.

## Specific aspects of Portuguese legislation

When available, the laws that regulate driving under the influence of psychoactive substances vary among countries. One typical difference is the accepted limit of the BAC for someone to be charged. Thresholds ranged from the limit of detection (zero tolerance) to 0.8 g/L (i.e. 0.08%). The website https://apps.who.int/gho/data/view.main.54600 of the World Health Organization compiles BAC limits by country. In Portugal, for the interpretation of analytical results, a driver or pedestrian who has a BAC equal to or greater than 0.5 g/L in the general regime or who, after medical examination carried out in accordance with the terms set out in the Highway Code and complementary legislation, is considered to be under the influence of alcohol (article 81 of the Decree-Law No. 44/2005, of February 23). However, Law No. 72/2013 of 3 September made an amendment to the Highway Code, creating special conditions for some drivers in its article 122. Thus, the BAC limit is 0.2 g/L for drivers on probation (e.g. driving license for <3 years), drivers of emergency or urgent service vehicles, public and emergency transports, or those transporting dangerous goods. In these cases, the legislation considers it to be a serious offense if the BAC is between 0.2 and 0.5 g/L. A value between 0.5 and 0.8 g/L is considered to indicate a very serious offense. A BAC equal to or greater than 1.2 g/L is considered to indicate a crime and punishable by up to 1 year in prison. Therefore, in opposition to other psychotropic substances, such as cannabinoids, amphetamines, cocaine, and opiates [5], for which the current legislation does not define concentrations or cut-offs, BAC is defined to assess the state of influence. However, these limitations should be considered when interpreting BACs collected from living individuals under sterile and aseptic conditions and not from postmortem blood, especially when dealing with samples collected from polytraumatized victims of road traffic accidents.

## Consequences of ethanol on driving and variability of toxicological response

The effects of ethanol, which primarily acts as a central nervous system depressant, have been shown to lead to a decrease in essential capabilities for safe driving [6, 7, 22–24] (Table 1). A lack of coordination, increased reaction time, or decreased psychosocial and motor capabilities are some of the consequences of driving under the influence of ethanol. This loss of capabilities, combined with behavioural changes that can lead to states of euphoria and disinhibition, indicates that drivers under the influence of alcohol have a much greater risk of being involved in road accidents due to their decreased ability to perform a task [7]. While there is no definitive consensus on the specific BAC threshold that signifies cognitive impairment in a driver, various studies have proposed some quantitative relationships [8, 9]. For example, several studies have shown that the risk of a road accident increases exponentially with BAC [10]: (i) at least 1.4 times with a BAC of 0.5 g/L, (ii) at least 2.7 times with a BAC of 0.8 g/L, (iii) at least 8.7 times with a BAC of 1.2 g/L, (iv) at least 22.1 times with a BAC of 1.5 g/L, and (v) at least 51.0 times with a BAC of 1.8 g/L. Other studies have demonstrated that for every 0.02 g/L increase in BAC, the risk of road traffic accidents doubles [11]. The risk of fatality following an accident is also greater for drivers under the influence of alcohol [12]: (i) at least three times greater if the BAC is between 0.2 and 0.5 g/L, (ii) at least six times greater if the BAC is between 0.5 and 0.8 g/L, and (iii) at least 11 times greater if the BAC is between 0.8 and 1.0 g/L. In addition to these acute ethanol exposure relationships, more recently, it was demonstrated for chronic cases that elevated concentrations of carbohydrate-deficient transferrin may be related to an increased risk of alcohol-related traffic accidents and may be used in procedures to reinforce driving licenses upon confiscation for “drunk driving” [13, 14]. Finally, it should be noted that there is great interindividual variability regarding the state of influence, which varies with age, frequency of consumption, height, weight, diet/fasting, genetic polymorphisms, sex, and pharmacokinetic tolerance, resulting mainly from the induction of the cytochrome P450 isoform CYP2E1 [15–17]. Pharmacodynamic tolerance is related mainly to the downregulation of receptors such as the N-methyl-D-aspartate (NMDA) receptor and GABA_A_, pathological conditions, and interactions with other substances. The analysis of each factor is outside of the aim of the review, and readers are encouraged to follow specific mechanistic and pharmacological pharmacokinetic and pharmacodynamic studies [15, 16, 18]. Specifically, regarding the interactions of ethanol with other substances, the following can influence either the psychoactive effect or BAC [19–21]: (i) pharmacodynamic interactions typically occur with other central nervous system depressants, and (ii) pharmacokinetic interactions, which seem to be less common than the previous interactions, are still not negligible: (a) antacids such as ranitidine and cimetidine (H_2_ antagonists) delay gastric emptying; (b) opioids also delay gastric emptying; (c) acetylsalicylic acid inhibits gastric alcohol dehydrogenases (ADH); (d) metronidazole inhibits ADH and aldehyde dehydrogenase (ALDH) and, consequently, their entire oxidative metabolism; (e) cocaine reduces BAC since cocaethylene is formed; and (f) ethanol interacts with paracetamol, stimulating the production of N-acetyl-p-benzoquinone imine (NAPQI) and carcinogenic metabolites of aflatoxin B_1_.

**Table 1:** Main effects of ethanol that may impair driving, which are more evident as the blood alcohol concentration increases [6, 7, 22–24].

No	Main effects
1	Decreased visual field
2	Diplopia (i.e. double vision; perception of two images from a single object) and impairs binocular visual acuity
3	Impaired night and stereoscopic (i.e. evaluates distance to objects) vision
4	Drowsiness and fatigue
5	Motor and sensory incoordination
6	Decreased reflexes, vigilance, and awareness of obstacles, increasing the reaction time
7	Euphoria and disinhibition
8	Risk of fatality due to suppression of vital functions
8	Memory lapses, amnesia (“blackouts”) and loss of consciousness
9	Tremors
10	Extreme confusion with very intense hallucinations
11	Increased mean speed, deviation of the lateral position, extended distance traveled outside the lane, and a higher frequency of collisions

## Factors that can influence antemortem ethanol analytical results

It is important to highlight several aspects to pay attention, dependent on whether we are considering a breath or a blood sample.

### Breath samples

Concerning breath samples, it is crucial to be mindful that the breath alcohol concentration (BrAC) relates more to the concentration of ethanol in arterial blood than to that in venous blood (typically collected from the median cubital vein) [25, 26]. In fact, during absorption, the BAC increases in the portal circulation, then in the right ventricle, pulmonary arteries, pulmonary capillaries (and it is from these that ethanol is eliminated in the alveolar air), pulmonary veins, and systemic arterial circulation before being distributed to the tissues. Only then does the BAC increase in venous blood, which is the sample that is taken from living persons.

The quantification of BrAC is possible since the concentration of ethanol in exhaled air is in equilibrium with the concentration in the blood of the pulmonary capillaries [27, 28]. For the purposes of applying the provisions of the Portuguese Highway Code, the conversion of BrAC values into BAC is based on the principle that in 1 mL of blood, there is an amount of ethanol equivalent to 2 300 mL of exhaled air (1 BAC = 2 300 BrAC). This relationship is called the blood-to-breath or partition relationship and permits the quantification of BAC based on BrAC. This conversion ratio is defined for each country and is not entirely specific in the scientific literature; indeed, this ratio is influenced by physiological variables, resulting in different reported ratios ranging from 1 300:1 to 3 100:1 [29–31].

The presence of residual alcohol in the oropharynx resulting from recent ingestion, vomiting or regurgitation of gastric contents, administration of cough medicines, mouthwash, or breath sprays may occasionally lead to higher values in exhaled air [32, 33]. To prevent the result from being influenced by ethanol still present in the mouth, a minimum interval of 15 min should be used from the time of last alcohol intake, and even if possible, the mouth should be rinsed with water before the test is performed [34]. This protocol is already implemented in certain jurisdictions, and during that period, officers must take precautions to ensure that the individual being tested does not consume any liquids, burp, regurgitate, or engage in any activities that could introduce ethanol into the mouth, potentially leading to inaccurate BrAC results.

The breathing pattern immediately preceding a breath test can also impact the measured BrAC [35–37]. Hyperventilation, achieved through rapid breathing for either 20 or 45 s before exhalation, resulted in a decrease in BrAC by 11%–12%. Conversely, hypoventilation was observed to increase BrAC by 7%–15% compared with the control group [35, 36, 38]. Owing to biological uncertainties, an individual who fully exhales their available breath volume is more likely to register a higher BrAC than a person with the same BAC who exhales only half of their available breath volume. This assumes both individuals share the same BAC, sex, lung volume, and body temperature.

### Blood samples

With respect to blood samples, collection is typically performed from antecubital fossa veins of the forearm [25, 26]. Differences exist in ethanol concentrations measured in plasma, serum, or whole-blood samples. In other words, ethanol levels in plasma, serum, and whole blood are not equivalent within the same person. Because ethanol is uniformly distributed throughout body water [39] and since the water content is greater in plasma and serum than in whole blood (94.2% in plasma to 84.8% in whole blood) due to the presence of figurative blood elements, the average concentration of alcohol in serum and plasma is ~1.09–1.18 times greater than that in BAC [40–44]. Determining the haematocrit levels can assist in rectifying variances, as an elevation in haematocrit (indicative of a rise in red blood cell count) along with a reduction in water volume leads to an increase in BAC. For legal purposes, it has been advised that the concentration of ethanol in plasma or serum measured at hospital laboratories be divided by a factor of 1.2. Even so, this practice aims to provide a conservative estimation of BAC [45]. The ethanol concentrations measured in serum or plasma are unaffected by the haematocrit if there is an extreme shift, as occurs in polycythemia, severe anaemia, or haemorrhage [40, 46].

Improper skin disinfection can serve as a potential source of contamination, introducing microorganisms from the skin microbiome [47]. Thus, ethanol may be more easily produced in the antemortem postsampled blood inside a test tube if a fermentation inhibitor is not present. One of the most interesting and recent studies focused on the *in vitro* alleged production of ethanol demonstrated that this analyte was not produced in blood tubes (collected following standard procedures such as disinfection of the skin and use of pre-evacuated tubes) after storage, regardless of the storage time, temperature, or the preservative addition [48]. Since blood microbiological contamination seems to be a trigger for ethanol production, forensic microbiological analyses will certainly help to exclude any possible source of endogenous ethanol. Irrespective of the inclusion of an inhibitor, a nonenzymatic oxidation of ethanol to acetaldehyde may take place, and the kinetics of this process is influenced by factors such as storage temperature, matrix oxygenation, and haemoglobin concentration [49–51].

The “after-drinking” scenario is another issue reported in the literature in cases related to driving under the influence of ethanol. These cases are referred to as the hip flask defence, in which following a motor vehicle accident, the driver evades law enforcement, and is later apprehended with a positive/illegal BAC [52, 53]. The individual then claims that the elevated BAC is a result of alcohol consumption after the incident. This type of defence tactic is completely ruled out in jurisdictions that consider consuming ethanol within a certain period after driving to be illegal.

The absorption of ethanol through the skin from antiseptics or hand sanitizers has not been demonstrated to elevate BAC [54, 55]. In experimental settings, significant ethanol contamination was registered during sampling when the needle was suctioning while in direct contact with alcoholic antiseptic solutions (e.g. pressing the soaked swab to control bleeding while withdrawing the needle) (e.g. pressing the soaked swab to stop bleeding while withdrawing the needle) [56, 57]. Otherwise (e.g. without pressing the soaked swab on the site of phlebotomy), with pre-evacuated tubes, there was no contamination, even when excess antiseptic was used (i.e. 2 mL) or when ethanol was not allowed to dry off (i.e. waiting for 5 s) [56, 58]. However, this blood contamination is erratic and unpredictable (e.g. depending on the degree of swab squeezing), and values ranging from 0.005 to 6.0 g/L have been reported [59, 60]. The European Federation of Clinical Chemistry and Laboratory Medicine (EFLM) guidelines do not ban alcoholic antiseptics for BAC requests but advise allowing for adequate time for drying before venipuncture [61]. Moreover, congener alcohol absorption from the use of hand sanitizers or occupational exposure was also detected [62].

Alcoholic fruit brandies and liqueurs included in chocolates were found to contain relevant concentrations of ethanol and congeners [63]. Studies focusing on the influence of chocolates administration and alterations of BAC were not yet performed, but it is reasonable to expect any legal repercussion. Certain pathological conditions, such as endogenous antemortem production due to microbial fermentation in the intestine (i.e. “auto-brewery” syndrome), can predispose individuals to a “perfect storm” [64].

Finally, it is important to pay attention to the two methods and tubes commonly used for venous blood collection for forensic purposes. When blood is collected using a “syringe and a needle”, an additional step is required, i.e. transferring the collected blood from the syringe into a clean, dry tube for subsequent transport to the laboratory for analysis. On the other hand, “pre-evacuated grey-top tubes” for BAC analysis for forensic purposes provide a more convenient method for collecting venous blood. These tubes are sold with an expiration date, which ensures the integrity of the vacuum seal, and they typically contain a mixture of sodium fluoride (NaF, 100 mg) as the preservative and 20 mg of potassium oxalate as the anticoagulant for 10 mL of capacity [65–67]. Although it is recommended that collecting tubes should be filled to reduce leakage by evaporation, the loss of ethanol does not seem to be influenced by the type of pre-evacuated tube used, whether it is made of glass or plastic [66, 68]. Indeed, when the sodium or potassium fluoride/oxalate tube is partially filled so that the sodium or potassium fluoride reaches a final concentration of 2% or 5% w/v, the bias produced at room temperature by the headspace vent was approximately −3.0% and −9.0%, respectively [69, 70]. This bias is attributed to the “salting-out” effect of the concentrated sodium or potassium fluoride in the matrix that increases ethanol evaporation into the headspace and consequently decreases the BAC [69, 70].

## Factors that can influence postmortem analytical results

The interpretation of the results of postmortem BAC is much more complex than that of antemortem blood, especially in cases of polytraumatized victims or those in a high state of decomposition. Moreover, in living individuals, blood is collected under sterile conditions, a fact that is not possible in postmortem samples since contamination most likely already occurred at the time of collection. Therefore, pragmatically considering two particularly important aspects, the risk of an artificial increase in BAC due to postmortem ethanol production or redistribution, is important.

### The risk of postmortem ethanol production

In the first hours after death, intestinal bacteria migrate to the portal venous system and, after ~6 h, contamination of systemic vessels already happen [71]. During the first 24 h after death, intestinal bacteria spread throughout the body, meaning that at the time of autopsy/blood collection microbial contamination is inevitable [72, 73]. Additionally, shortly after death, microbes of the respiratory system and from the environment access surrounding tissues. Moreover, many species of bacteria, yeasts, and molds can produce ethanol and other volatiles from various substrates [74]. Therefore, in routine postmortem toxicology casework, it is commonly registered low BACs (i.e. 0.1–0.33 g/L) even when there was no evidence suggesting that the deceased had consumed any ethanol during life [3]. Therefore, many toxicology laboratories only report a positive BAC when the toxicological result is higher than 0.1 or 0.2 g/L [75]; below these levels, BAC is reported as “ethanol not detected”. If the cadaver is decomposed, a conservative and recommended approach to increase the confidence of the result is to subtract 0.5 g/L from the mean analytical result [75].

Endogenous ethanol production occurs by the anaerobic action of microorganisms on endogenous substrates, especially carbohydrates such as hexose glucose. The most commonly used pathway for glucose metabolism is the Embden–Meyerhof–Parnas glycolytic pathway, which generates two molecules of ethanol and two molecules of carbon dioxide from a glucose molecule. Therefore, and theoretically, if the glucose concentration in blood or urine is 1 g/L (i.e. (1g/L)/(180.156 g/L) = 0.00555 mol/L), simple calculations showed that glycolysis and fermentation would result in a BAC of 0.5 g/L (i.e. 0.005 55 mol/L glucose × 2 moles of ethanol × 46.068 g/mol = 0.511 g/L of ethanol) [76, 77]. However, since postmortem blood glucose concentration and the influence of several variables (e.g. trauma, diabetes status, possibility of hyperglycemia, and postmortem interval) at the time of autopsy are unknown, it is difficult to determine how much ethanol might theoretically be produced as consequence of microbial activity. The main microorganisms involved are *Candida albicans*, *Clostridium perfringens*, *Clostridium sporogenes*, *Streptococcus faecalis*, *Escherichia coli*, *Klebsiella pneumoniae,Lactobacillus* sp., and *Proteus vulgaris* [78, 79]. Moreover, ethanol can also be generated through other pathways, such as mixed acid and butanediol fermentation carried out by enterobacteria; from glycerol (a product of lipid hydrolysis or carbohydrate metabolism) by enterobacteria, clostridia, and yeasts [74]; and from mannitol administered antemortem [80].

Elevated environmental temperatures, duration of storage [81, 82], terminal severe hyperglycemia, septicemia, lesions produced by piercing, cutting, blunt instruments, mutilations, *etc.*, which represent orifices through which environmental microorganisms can enter, represent fertile conditions for the postmortem production of ethanol and therefore the source of interpretative artefacts [83]. The risk of ethanol production in bodies autopsied within 24 h after death is not significant, and this time window increases in causes of death related to hypothermia [75, 76]. The abundance of substrates during the early postmortem interval creates more favourable conditions for ethanol production; then, the BAC peaks and decreases gradually over time [74, 84]. Furthermore, it has been proposed that the findings should be adjusted considering the decrease in water content of blood as the postmortem interval extends. This adjustment would involve comparing the water content with that of fresh blood from living subjects, typically ranging between 78% and 82% w/w water [85, 86]. As the microbiological contamination of cardiac blood is more rapid compared to other blood anatomic compartments, and given that glycogen-rich tissues such as the heart, liver, and lungs provide higher substrate concentrations for postmortem ethanol formation, this may explain the increased BAC in cardiac blood compared to peripheral (e.g., femoral) blood [87].

The recommended precautions to decrease (but most likely not to eliminate) microbial activity after sampling (and consequently decrease ethanol and other volatile formations) include blood collection for tubes with appropriate concentrations of an enzyme inhibitor preservative (e.g. 1%–2% w/v sodium or potassium fluoride) and storage under correct refrigerating conditions (4°C) [88]. Obviously, this procedure does not avoid the biosynthesis of ethanol that already occurs in the body before collection.

Under certain circumstances, BAC postmortem results should be compared with the analysis of other fluids, especially those that are more resistant to microbial contamination, such as the vitreous humour and urine [26, 89, 90]. Since ethanol is distributed throughout the total body water compartment, at equilibrium, greater concentrations of ethanol are reached in fluids such as vitreous humour (contains 93%–99% w/w water) and tissues with higher water content in comparison with whole blood (contains 78%–82% w/w water) or plasma/serum (contains 91% water) [75]. Excluding the traumatic cases of the eyes, the presence of ethanol in the vitreous humour is a good indicator of antemortem consumption since it is anatomically isolated, protected, and distant from intestinal microorganisms. With respect to urine, excluding antemortem cases of urinary tract infection and traumatisms of the pelvis and surrounding structures, this fluid is more resistant to contamination by postmortem microorganisms and does not contain glucose except in diabetic patients. Thus, the presence of ethanol in this sample is more likely to suggest exogenous exposure. Finally, drowning bodies pose additional challenges due to potential fluid dilution, decomposition, and an increased risk of microbial synthesis of ethanol [91].

### The specific case of postmortem redistribution

Postmortem redistribution is a great challenge in postmortem toxicology since the corpse is not a static postmortem entity as far as the distribution of xenobiotics and endobiotics is concerned. This phenomenon depicts the movement of compounds between organs, between vascular compartments, and between vascular compartments and organs and *vice versa* and depends on several factors. Regarding ethanol, the passive diffusion from the gastric contents (i.e. not yet metabolized or absorbed), or from the respiratory tract as a result of aspiration of vomit, continues after death [92]. This may, in addition to the risk of increased production described above, result in an artificial increase in BAC when samples are obtained from the heart, pericardial sac, large thoracic vessels, thoracic and abdominal cavities in comparison with other peripheral vessels [93–95]. Moreover, removal and transport of the body may increase the risk of postmortem redistribution [93], with some studies showing unacceptable variation in BAC according to the sampling site [96]. For all these reasons, in postmortem toxicology, sampling of peripheral blood from the femoral, subclavian, or jugular veins or arteries is recommended [26]. Arterial or venous peripheral blood may also produce different BACs. During absorption, the arterial BAC is greater than the venous concentration; after absorption, the BACs tend to equalize; and in the postabsorptive phase, the venous BAC tends to exceed the arterial one [30, 97].

Finally, although the most frequent scenario is a postmortem increase in BAC, a decrease in BAC can also occur. Indeed, reanalysis of stored frozen samples (−20°C) for up to 12 months is needed if a confirmatory analysis is requested, as these samples tend to produce <8% of the BAC even when preservatives containing fluoride are present [98]. Moreover, the possibility of a decrease in BACs was also demonstrated for two species of bacteria, *Pseudomonas* sp. (probably *Pseudomonas putida*) and *Serratia marcescens*, when isolated from contaminated samples [99].

## Possibilities exist to differentiate exogenous ethanol sources from endogenous sources

In view of the various constraints described above, several studies have proposed strategies to differentiate exogenous/ingested ethanol from that resulting from endogenous production or postmortem redistribution processes. Among them, the following strategies are outlined:

- **In the comparison of ethanol concentrations between several different samples** (i.e. blood, vitreous humour, and urine) taken from the same cadaver, different results between the different samples (e.g. positive ethanol in blood and negative ethanol in vitreous humour and urine) most likely indicated a case of endogenous ethanol production. Ratios between different matrices have been proposed, but their use is very limited given the multiple intercurrent factors [90, 100–102].
- **The presence of other volatile compounds of low molecular weight** in the toxicological results, including 1-propanol, 1-butanol, isobutanol, 2-methyl-1-butanol (isoamyl-alcohol), 3-methyl-2-butanol (amyl-alcohol), acetone, and acetaldehyde, in postmortem samples has been correlated with putrefaction and microbial activity both in corpse presampling or *in situ* postsampling. Since these analytes are produced together with ethanol by microorganisms, they suggest endogenous ethanol production, and some cut-off criteria have been proposed. Therefore, routine inspection of gas chromatograms used for analysis of suspected peaks with the expected retention times of those volatiles is important when the corpse shows signs of decomposition. However, some scientific literature suggests that several of these alcohols (e.g. 1-propanol) may also be present in beverages [52, 79, 103, 104], especially in distilled spirits, unlike in fermented spirits. Indeed, distillation has the dual effect of enhancing ethanol concentration and influencing the proportion of congeners produced during fermentation. Through the process of distillation, ethanol and volatile congeners are separated based on their boiling points. Congeners with boiling points similar to or lower than ethanol (such as methanol) are also subjected to distillation. Another study confirmed the endogenous formation of 1-propanol and methanol after consumption of an alcoholic beverage and that 1-propanol formation increases with increasing ethanol dose [105]. Notably, in addition to ethanol, 1-propanol is the most abundant alcohol detected in the postmortem period. Therefore, while using 1-propanol as the internal standard in the antemortem analysis of BAC, this three-carbon alcohol is less appropriate as an internal standard for the analysis of postmortem blood because it is also produced [73, 106]. In cases of concurrent existence of two or more higher alcohols in a postmortem blood sample, the ethanol origin of this sample should be questioned [107]. Assessing the presence of methanol in blood samples is challenging, as it may result from pectin fermentation not only in the beverage itself but also from endogenous formation [108].
- **Analysis of EtG and EtS** as direct, nonvolatile metabolites of ethanol has revealed relevant clinical and forensic applications [109]. EtG and EtS are phase II metabolites of ethanol that are catalysed by the enzymes of the UDP-glucuronosyltransferase (UGT; utilizes UDP-glucuronic acid as a cofactor) and by sulfotransferase (SULT; uses 3′-phosphoadenosine-5′-phosphosulfate as a cofactor) superfamilies, respectively. Less than 2% of the ethanol ingested is metabolized through this nonoxidative route [110]. Within just 1 h of consumption of low amounts of ethanol, EtG and EtS are already positive in urine, and, due to their longer half-life, they are still present in this matrix even when ethanol is negative [111]. Therefore, EtG and EtS are useful biomarkers for documenting ethanol exposure and for monitoring abstinence [112–114]. In other words, negative results indicate that ethanol was not ingested before death [115–117]. However, false-positive EtG results may occur if the collected urine contains bacteria (e.g. *E. coli*) and ethanol, which might be produced from glucose (e.g. glycosuria in diabetic patients) and yeast by alcoholic fermentation [118]. On the other hand, false-negative EtG results may also be recorded for urine samples (mainly if the sample is transported and stored improperly without cooling) since this metabolite is sensitive to hydrolysis by β-glucuronidase, which is present at high levels in *E. coli* and is a common cause of urinary tract infection [75–77]. Therefore, refrigeration or frozen storage is recommended to improve EtG stability in these samples [119]. Neither postsampling formation nor degradation has been documented in urine samples for EtS [120]. Some studies have reported EtS biodegradation only under high-density bacterial conditions [120]. Finally, pathological conditions such as Gilberts syndrome and genetic polymorphisms in UGT and SULT may explain inter- and intraindividual differences in EtG and EtS levels [15, 121].
- **Analysis of other nonoxidative metabolites of ethanol, such as PEth**. This compound has been detected in the blood of individuals dependent on the ethanol, even up to three weeks after withdrawal [114, 122]. Moreover, the PEth concentration is strongly correlated with the amount of ingested ethanol [122]. These findings show that the PEth concentration may constitute a promising biomarker for detecting ethanol abuse and monitoring abstinence [123]. False PEth-positive results have been reported since it can be formed *in vitro* in blood samples containing ethanol at −20°C and at room temperature [124, 125]. The presence of PEth in the blood is considered proof that ethanol was ingested, and not produced by anaerobic formation *via* pyruvate and fermentation [126].
- **Ethanol consumption can alter the concentration of two major serotonin metabolites, 5-hydroxytryptol (5-HTOL)/5-hydroxyindoleacetic acid (5-HIAA)** [127]. The urine 5-HTOL/5-HIAA ratio is usually very low in abstemious individuals and increases during hepatic ethanol metabolism [128].

## Conclusions and future perspectives

The evaluation of states of influence by psychotropic substances while driving is a complex clinical and forensic issue [5]. The success of this approach will always be much dependent on the preanalytical aspects regarding sample collection [26]. The forensic toxicologist is challenged to provide scientific evidence to distinguish the source of ethanol through antemortem ingestion or other sources, such as microbial production and postmortem redistribution. As previously mentioned, it is difficult to determine whether someone who is “dead sober or dead drunk” [129]. For the correct interpretation of the blood alcohol results, it is necessary to (i) evaluate the physical integrity of the body (e.g. if polytrauma or the presence of haemorrhage), (ii) estimate the postmortem interval according to the putrefactive signs, (iii) document the site of blood collection (e.g. whether from the cardiac, thoracic or abdominal cavities, or from a peripheral site such as the vein or femoral artery), (iv) interpret the results of alternative samples such as vitreous humour and urine and compare the results with BAC, (v) perform forensic microbiology studies to assess what species of microorganisms (i.e. whether an ethanol producer or catalyst) may have colonized the sample, (vi) evaluate the possibility of postmortem redistribution due to ethanol from the stomach not yet absorbed, (vii) ensure that preservatives were effectively used in the collection tubes and that optimal temperature conditions of shipment for analysis and storage were respected, (viii) ensure that embalming fluids containing ethanol were not used, and (ix) determine the type of sampling container used (i.e. glass/plastic/closure/air space).

Finally, the possibility of auto-brewery syndrome should not be disregarded; therefore, a clinical history is fundamental [64]. While rare, unstudied, and often underreported, patients experiencing this syndrome exhibit symptoms of inebriation and may face medical, social, and forensic consequences associated with alcoholism, such as arrests for drunk driving. The pathophysiology of auto-brewery syndrome is thought to be linked to fungal-type dysbiosis in the gut, leading to the fermentation of certain carbohydrates into ethanol [64]. Thus, to increase the robustness of the toxicological report, a written explanation and discussion about the possibility that some BACs may have resulted from cadaveric phenomena should always be considered. As previously emphasized, “not everything that can be counted counts and not everything that counts can be counted” (attributed to Albert Einstein).
